# First results of the French OCTOPUS survey among festival attendees: a latent class analysis

**DOI:** 10.1186/s12954-023-00770-5

**Published:** 2023-03-29

**Authors:** Marion Istvan, Vincent Bresdin, Marie Mainguy, Pauline Laigo, Marie Grall-Bronnec, Vincent Eudeline, Jean-Emmanuel Guillet, Manon Guillo, Laurent Babonnaud, Pascale Jolliet, Benoit Schreck, Caroline Victorri-Vigneau

**Affiliations:** 1 Nantes Université, CHU Nantes, Centre d’Evaluation et d’Information sur la Pharmacodépendance-Addictovigilance (CEIP-A), Service de Pharmacologie Clinique, F-44000 Nantes, France; 2 Nantes Université, Univ Tours, CHU Nantes, CHU Tours, INSERM, MethodS in Patients-centered outcomes and HEalth Research, SPHERE, F-44000 Nantes, France; 3 Nantes Université, CHU Nantes, UIC Psychiatrie et Santé Mentale, F-44000 Nantes, France; 4 OPPELIA, Association de Prévention et de Soin en Addictologie, Nantes et St Nazaire, France; 5 Techno + Nantes, Association de Santé Communautaire en Milieu Techno, Nantes, France; 6 AIDES Nantes, association de soutien et accompagnement des personnes, Actions de prévention et de réduction des risques du VIH/SIDA et des hépatites, Nantes, France

**Keywords:** Festivals, Party scene, Illicit drugs, Psychoactive substances, Polysubstance use, Latent class analysis

## Abstract

**Background:**

Illicit substance use has constantly evolved over the years, particularly in the party scene. Monitoring these changes is essential to adapt harm reduction strategies. The OCTOPUS survey was implemented to enhance knowledge on drug use at music festivals. The objective of the work presented here was to describe drug use and to characterize profiles of substance use in music festival attendees.

**Methods:**

OCTOPUS was a cross-sectional survey carried out during 13 various music festivals (dub, eclectic and electronic music) in the Loire-Atlantique department (France) from July 2017 to July 2018. Participants were festival attendees. Data were collected by trained research staff using a face-to-face structured interview. We analysed the use of illicit drugs in the last 12 months to describe the prevalence of use and to characterize the profile of substance use using a latent class analysis.

**Results:**

In total, 383 festival attendees were included. Of 314 (82%) participants who reported drug use, the most reported drugs were cannabis, ecstasy/MDMA and cocaine. We identified two profiles of drug use: (i) a “no/low polysubstance use” profile mainly characterized by the use of “classic” stimulants (ecstasy/MDMA, cocaine) and (ii) “moderate/extensive polysubstance use”, with high probabilities of “classic” stimulants use and especially other drugs reported: speed, ketamine, new psychoactive substances (NPSs).

**Conclusion:**

We observed frequent polysubstance use in festival attendees. Harm reduction should be targeted at the increased risk of toxicity linked to polysubstance use, and the reduction in harm caused by particular drugs (ketamine, NPS, speed) could be further strengthened.

## Introduction

The use of illicit substances is very common in France but also in Europe as a whole. In Europe, the proportion of 15- to 64-year-old persons who use illicit drugs (at least one experience in their lifetime) is estimated at 29% (83 million Europeans) and is especially common in young adults [1]. In France, the proportion of persons using illicit drugs is even higher than that in Europe. For example, in 2017, the lifetime prevalence of cannabis use is 45% among French people aged 15 to 64 [1]. In recent years, the frequency of illicit substance use has increased. In almost 20 years, in France, the proportion of persons who used cannabis in the previous year increased from 8 to 11% and fivefold for cocaine among the general population [2]. The panorama of illicit drugs used is also changing. Since the 2010s, new substances named “new psychoactive substances” (NPSs) emerge. These substances are designed to mimic the effects of illicit substances used for certain drugs with very close chemical structures and circumvent drug regulation. Different types of NPSs exist according to their molecular structure, and the most frequent are synthetic cannabinoids and cathinones [1, 3]. The number of different NPSs on the market is dramatically increasing in recent years [3–5]. Since 2015, 400 different NPSs are available every year in Europe, and a total of 830 different NPSs are under surveillance in 2020 [1].

The quantitative and qualitative changes in illicit substance use are concomitant with easier accessibility and availability of the products (net and social networks) [5]. In addition, they are particularly observed in the party scene (festivals, rave parties, nightclubs, etc.), where a normalization of illicit drug use is observed [2, 6]. Indeed, the party scene is a key place for illicit drug use (experimentation and recurrent use) [7–10], and especially frequently the place of polysubstance use [11]. Polysubstance use among partygoers is shown to be associated with frequent acute adverse effects (memory impairment, tachycardia, bad mood after use, insomnia) [12, 13] due to possible summation of effects or interactions [14, 15]. A higher frequency of emergency treatment episodes and accidents is also observed in persons with polysubstance use [12]

Monitoring illicit drug use in the party scene is essential to adapt harm reduction strategies. This monitoring can be performed using different study designs [7]: wastewater-based drug epidemiology to monitor the type and quantity of drugs [16–20] or prevalence studies in festival attendees using questionnaires [7, 11, 21–26]. These studies provide key results about circulating drugs (the identification of new drugs and evolution of known ones), but they do not provide information about the profiles of illicit drug use taking into account polysubstance use. This limitation is all the more true at festivals and in nightclubs, where polysubstance use is known to be frequent [11]. To our knowledge, only a few studies have analysed consumer profiles of illicit drug use in the party scene [13, 27–29] and none have focused on the French population.

France is one of the only European countries with a dedicated national system of surveillance of abuse and dependence on psychoactive substances [named Addictovigilance] that relies on a network of 13 Centres for Evaluation and Information on Pharmacodependence–Addictovigilance (CEIP-A). One of the missions of the Addictovigilance network is to collect data and assess abuse and dependence potential, allowing us to assess the evolution of substance use and the appearance of new substances. Indeed, in France the declaration by health professionals of cases of abuse, dependence and/or consequences related to substance use is mandatory [30]. However, this surveillance system has some limitations in monitoring drug use in the party scene because it includes only information on: (i) persons who use substances and integrated in the health care system and (ii) cases of abuse or dependence. To enhance knowledge about drug use in the party scene, a large survey named OCTOPUS (Observation of ConsumpTion Of Psychoactive substance Use in music festivalS) was established by the CEIP-A of the French Pays de la Loire region and by centres or associations dedicated to drug harm reduction, prevention or care.

The objective of the study presented here was i) to describe the prevalence of drug use and ii) to identify and characterize the profiles of use among festival attendees of various musical events using data from the OCTOPUS survey.

## Material and methods

### Study design

OCTOPUS was a cross-sectional survey established by the CEIP-A of the French Pays de la Loire region and by centres or associations dedicated to drug harm reduction, prevention or care and funded by the Pays de la Loire regional health agency. The survey was monitored by a multidisciplinary steering committee of pharmacologists specializing in addictology and social workers from centres or associations for harm reduction addiction care and prevention. The survey was performed at 13 various music festivals (dub, eclectic and electronic music) in the department of Loire-Atlantique from July 2017 to July 2018.

### Participants

Participants were recruited according to the following inclusion criterion: age at least 18 years with a good understanding of French. Investigators met with festival attendees at random and offered them to participate in the study.

### Data collection

Data were collected using a face-to-face structured interview before 1:00 a.m. by recruited volunteers trained in addiction care or harm reduction strategies. The following data were collected: sociodemographic characteristics, substance use (alcohol, tobacco consumption and other psychoactive substances as an open question) and harm reduction (knowledge about harm reduction and access to prevention or care facilities).

In this work, we analysed the music type of the included festivals and the following sociodemographic data: age, gender, region of residence, employment status, education level, household type and the existence of a long-term disease or long-term drug treatment. Education level was categorized as follows: the first stage of primary education or secondary education (*collège*) corresponding to the International Standard Classification of Education (ISCED) 0 to 2, high school/baccalaureate diploma (ISCED 3–4) and higher education (ISCED 5 to 8). Regarding substance use, we analysed the frequency of tobacco and alcohol consumption, frequency of binge drinking (defined as 6 or more drinks on one occasion) and frequency of other psychoactive substance use in the past year. We characterized the use of other psychoactive substances using the number of different substances reported, the regularity of use (at least monthly), and problematic use among persons with regular use. Problematic use was defined as the presence of 3 criteria or more according to the Diagnostic and Statistical Manual of Mental Disorders, 4^th^ Edition, Text Revision (DSM-IV-TR) [31]. Regarding harm reduction, we analysed the search for information and the accessibility to individuals or structures involved in harm reduction.

### Ethics

The study was conducted in accordance with the 1964 Helsinki declaration and its later amendments. Informed consent was collected from each participant during the interview. Only anonymous data in the context of the regulated purposes of the Addictovigilance system were collected.

### Statistical analysis

The prevalence of persons using illicit drugs was first calculated using the number persons using illicit drugs in the past year divided by the number of participants. We then analysed the profiles of illicit drug use using latent class analysis (LCA) among persons using illicit drugs in the past year (except for alcohol and tobacco use). LCA is a probabilistic model to identify similar patterns of response to indicators (categorical variables such as drug use in the previous year, in our study). The goal is to identify the smallest number of groups of participants, named classes, that best describe the variation in responses to the indicators. In our work, a class included participants with similar drug use patterns. In the LCA model, we included drug use with a prevalence of at least 5%, except for cannabis, for which the very high frequency made the classes less interpretable. Two parameters of LCA were interpreted: the probability of class membership, which corresponded to the prevalence of the class, and the probability of responses to the categorical variables (illicit drug use) in each class, which corresponded to the probability of the indicator in the class. Bivariate residuals were taken into account in the context of the local independence assumption. The best number of classes was defined according to the smallest Bayesian information criteria (BIC) value, information criterion performing the best for deciding the number of classes [32]. To characterize the characteristics of the classes, each participant was classified in the class for which the probability of membership was the highest. We then described and compared sociodemographic, consumption characteristics and responses regarding harm reduction using Student’s t test and the Chi-square test or Fisher’s exact test.

Analysis was performed using SAS® software version 9.4 (SAS Institute Inc.) and Latent Gold® software version 5.1 (Statistical Innovations, Belmont, MA).

## Results

### Description of the study population

In total, 383 participants attended different types of music festivals (47%, *N* = 180 in electronic music festivals, 35%, *N* = 134 in eclectic music festivals and 18%, *N* = 69 in dub music festivals). The participants were mostly young (68%, *N* = 260 under 30 years old), male (65%, *N* = 249), and employed (64%, *N* = 237) and had a high education level (64%, *N* = 244). Overall, 10% of the participants reported long-term disease and long-term drug treatment (respectively, *N* = 45 and *N* = 37). A large majority of participants reported tobacco and alcohol consumption (81%, *N* = 309 and 97%, *N* = 373, respectively) (Table 1).

**Table 1:** Description of the characteristics of the study population (*n* = 383 participants)

Variable	
Musical genre of the festival, *n* (%)	
Electronic	180 (47.0)
Eclectic	134 (35.0)
Dub	69 (18.0)
Gender, *n* (%)	
Male	249 (65.2)
Female	132 (34.6)
Transgender	1 (0.3)
Age (years), *n* (%)	
18–24	143 (37.3)
25–29	117 (30.6)
≥ 30	123 (32.1)
Region of residence, *n* (%)	
Pays de la Loire	282 (73.8)
Bretagne	37 (9.7)
Other regions	63 (16.5)
Employment status, *n* (%)	
Employed	237 (63.5)
Student	89 (23.9)
Unemployed	34 (9.1)
Volunteer (association, civic service)	12 (3.1)
Retired	1 (0.3)
Education level, *n* (%)	
Higher education	244 (64.0)
High school/baccalaureate diploma	96 (25.2)
First stage of primary or secondary education (*collège*)	41 (10.8)
Household type, *n* (%)	
Living alone	166 (44.9)
Living as a couple	103 (27.8)
Living with their parents	45 (12.2)
Living with other people	56 (15.1)
Long-term disease, *n* (%)	45 (11.9)
Long-term drug treatment, *n* (%)	37 (9.9)
Tobacco consumption, *n* (%)	
Party use	36 (9.5)
Unknown or < 5 cigarettes/day	60 (15.7)
5–10 cigarettes/day	113 (29.7)
> 10 cigarettes/day	98 (25.7)
None	74 (19.4)
Alcohol consumption^a^, *n* (%)	
Party	146 (38.1)
Occasional	118 (30.8)
Moderate	71 (18.5)
High	38 (9.9)
None	10 (2.6)
Binge drinking^b^, number of times per month, *n* (%)	
Any	35 (9.2)
≤ 1 time	110 (29.0)
2–4 times	133 (35.1)
> 4 times	101 (26.7)

Missing values: < 1%: gender, region of residence, education level, tobacco consumption; 1–4%: Employment status, household type, long-term disease, long-term drug treatment, binge drinking

^a^Occasional: up to 1 unit of alcohol per day, moderate: up to 2 units of alcohol per day for females and 3 units of alcohol per day for males, high: more than 2 units of alcohol per day for females and 3 units of alcohol per day for males

^b^6 or more drinks on one occasion

### Prevalence of drug use

Among the 383 participants, 314 (82%) reported drug use in the last 12 months, and the most reported drugs were cannabis (63%, *N* = 243), ecstasy/MDMA (49%, *N* = 186) and cocaine (42%, *N* = 159). Other drugs were also reported but to a lesser extent: lysergic acid diethylamide (LSD) (18%, *N* = 67), magic mushrooms (11%, *N* = 42), ketamine (10%, *N* = 37), speed (8%, *N* = 32), NPSs (5%, *N* = 19), and poppers (4%, *N* = 17). The NPSs reported were mostly synthetic hallucinogens (10/19) and synthetic stimulants (7/19) (Table 2).

**Table 2:** Prevalence of the different psychoactive drugs used in the last 12 months in the total population (*n* = 383) and persons who use psychoactive substances (*n* = 314)

Psychoactive drug, *n* (%)	Number of persons who use drugs	Prevalence in the total population *N* = 383 (%)	Prevalence among the persons who use drugs *N* = 314 (%)
Cannabis, *n* (%)	243	63.4	77.4
Ecstasy/MDMA, *n* (%)	186	48.6	59.2
Cocaine, *n* (%)	159	41.5	50.6
Speed, *n* (%)	32	8.4	10.2
Poppers, *n* (%)	17	4.4	5.4
Other stimulants^a^, *n* (%)	15	3.9	4.8
LSD, *n* (%)	67	17.5	21.3
Magic mushrooms, *n* (%)	42	11.0	13.4
Ketamine, *n* (%)	37	9.7	11.8
Other hallucinogens^b^, *n* (%)	4	1.0	1.3
NPSs^c^, *n* (%)	19	5.0	6.1
Sedatives^d^, *n* (%)	19	5.0	6.1
Cannabidiol	1	0.3	0.3

*MDMA* Methylenedioxymethamphetamine, *LSD* Lysergic acid diethylamide, *NPSs* New psychoactive substances

^a^GBL (*n* = 2), crack (*n* = 2), Ritalin (*n* = 1), ephedrine (*n* = 1), caffeine (*n* = 1), amphetamine (*n* = 8)

^b^*Salvia divinorum* (*n* = 2), mescaline (*n* = 2)

^c^NPSs in detail: Synthetic stimulants (*n* = 7): mephedrone (*n* = 3), 3-MMC (*n* = 2), 4-MEC (*n* = 1), methylone (*n* = 1), ethylphenidate (*n* = 2). Synthetic hallucinogens (*n* = 10): DMT (*n* = 4), 5-APB (*n* = 3), methoxetamine (*n* = 2), 25C-NBOMe (*n* = 1), 25I-NBOMe (*n* = 1), 2C-P (*n* = 1), 2C-B (*n* = 2), 2C-E (*n* = 1), Bk-2C-B (*n* = 1), DOC (*n* = 1), deschloroketamine (*n* = 1). Synthetic opioids (*n* = 5): U-47700. Synthetic cannabinoids (*n* = 2)

^d^opium (*n* = 7), heroin (*n* = 5), methadone (*n* = 2), nitrous oxide (*n* = 2), tramadol (*n* = 2), Valium (*n* = 2), codeine (*n* = 1), Skenan (*n* = 1), Xanax (*n* = 1)

### Profiles of drug use

**Fig. 1:**
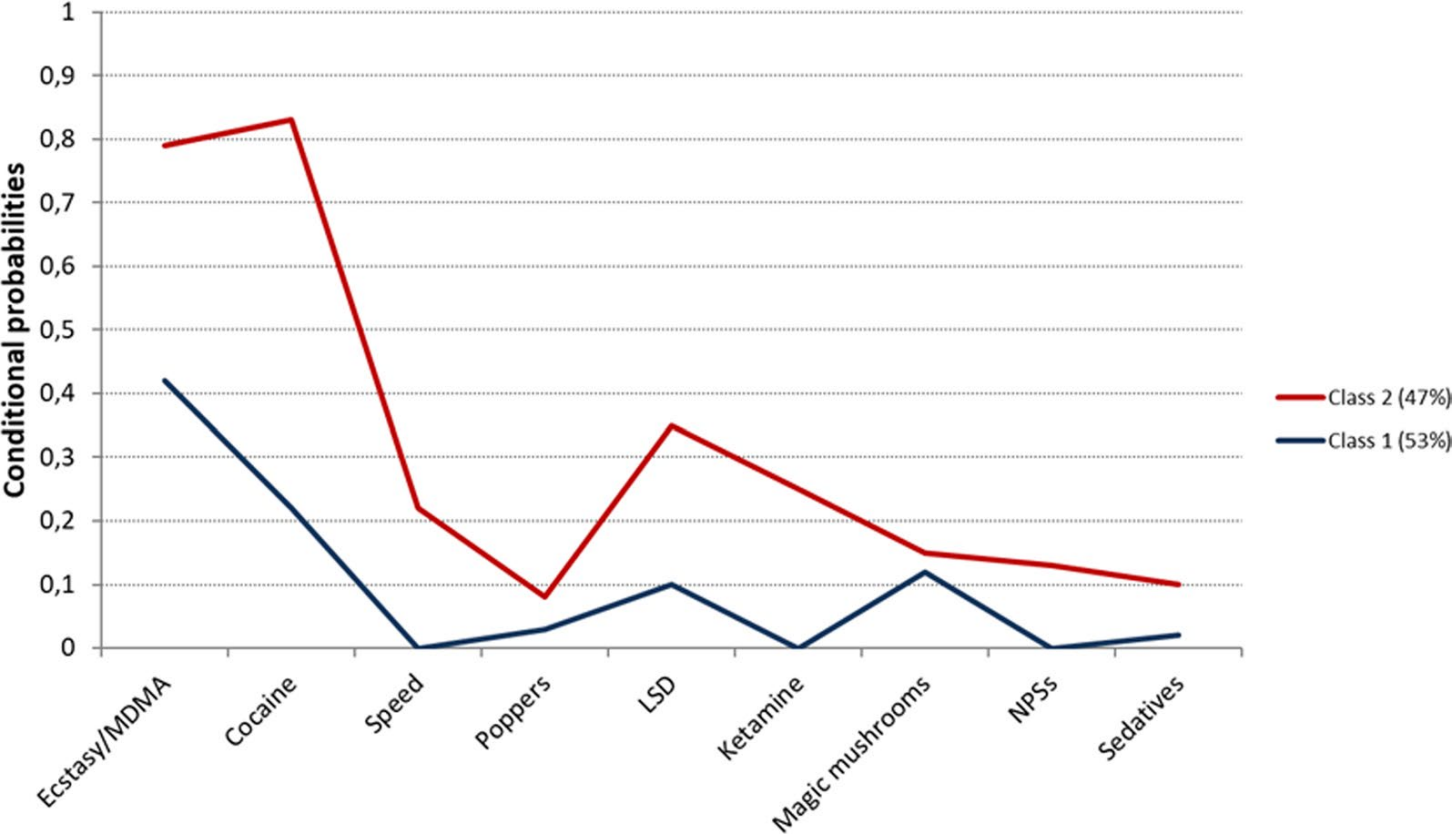
Conditional probabilities of drug use in the latent classes (*n* = 2). *MDMA* Methylenedioxymethamphetamine, *LSD* Lysergic acid diethylamide, *NPSs* New psychoactive substances

Two classes were identified with the best fit (BIC = 2261). Conditional probabilities are presented in Fig. 1 and Table 3.

**Table 3:** Description of estimated probabilities and sociodemographic and consumption characteristics in the two classes (*n* = 314)

	Class 1 *N* = 163 (53%)	Class 2 *N* = 151 (47%)	*p* value
Indicator, %			
Ecstasy/MDMA	42%	79%	
Cocaine	22%	83%	
Speed	0	22%	
Poppers	3%	8%	
LSD	10%	35%	
Ketamine	0	25%	
Magic mushrooms	12%	15%	
NPSs	0	13%	
Sedatives	2%	10%	
Characteristics of the classes, *n* (%) or mean (SD)			
Number of different psychoactive drugs reported	1.7 (0.8)	3.9 (1.5)	< 0.001
Cannabis use	138 (84.7)	105 (69.5)	0.001
Cannabis use only	63 (38.7)	0	< 0.001
Musical genre of the festival			0.034
Electronic	72 (44.2)	81 (53.6)	
Eclectic	61 (37.4)	36 (23.8)	
Dub	30 (18.4)	34 (22.5)	
Age			0.70
< 25 years	64 (39.3)	61 (40.4)	
25–29 years	48 (29.5)	49 (32.5)	
≥ 30 years	51 (31.3)	41 (27.2)	
Gender			0.580*
Male	115 (71.0)	103 (68.2)	
Female	46 (28.4)	48 (31.8)	
Transgender	1 (0.6)	0	
Household type			0.207
Living alone	73 (45.6)	62 (43.4)	
Living as a couple	44 (27.5)	30 (21.0)	
Living with their parents	21 (13.1)	19 (13.3)	
Living with other people	22 (13.8)	32 (22.4)	
Employment status			0.036
Employed	111 (69.8)	90 (60.8)	
Unemployed	9 (5.7)	21 (14.2)	
Student	39 (24.5)	37 (25.0)	
Education level			0.365
Higher education	99 (60.7)	92 (61.3)	
High school/*baccalaureate* diploma	41 (25.2)	44 (29.3)	
First stage of primary or secondary education (*collège*)	23 (14.1)	14 (9.3)	
Tobacco consumption			0.040
None	26 (16.1)	11 (7.3)	
Party use	16 (9.9)	14 (9.3)	
Unknown or < 5 cigarettes/day	32 (19.8)	20 (13.3)	
5–10 cigarettes/day	47 (29.0)	55 (36.7)	
> 10 cigarettes/day	41 (25.3)	50 (33.3)	
Alcohol consumption^a^			0.028
None	6 (3.7)	0	
Party	65 (39.9)	56 (37.1)	
Occasional	53 (32.5)	40 (26.5)	
Moderate	26 (16.0)	35 (23.2)	
High	13 (8.0)	20 (13.3)	
Binge drinking^b^, number of times per month			< 0.001
Any	17 (10.6)	4 (2.7)	
≤ 1 time	53 (32.9)	33 (22.2)	
2–4 times	60 (37.3)	51 (34.2)	
> 4 times	31 (19.3)	61 (40.9)	
Harm reduction, n (%)			
Do you get information about substances before using them?			0.04
Yes	118 (75.2)	126 (84.6)	
No	39 (24.8)	23 (15.4)	
Do you find the risk reduction materials easily accessible?			0.96
Yes	82 (56.2)	81 (55.9)	
No	64 (43.8)	64 (44.1)	
Do you find risk reduction information easily accessible?			0.80
Yes	99 (66.9)	95 (65.5)	
No	49 (33.1)	50 (34.5)	
Do you know any health professionals you can contact?			0.87
Yes	115 (74.2)	111 (75.0)	
No	40 (25.8)	37 (25.0)	
Do you know any social actors you can contact?			0.009
Yes	70 (44.6)	88 (59.5)	
No	87 (55.4)	60 (40.5)	
Do you know any harm reduction and/or prevention structures you can contact?			0.003
Yes	79 (51.0)	100 (68.0)	
No	76 (49.0)	47 (32.0)	

Missing values: < 1%: gender, region of residence, education level, tobacco consumption; 1–7%: Employment status, household type, long-term disease, long-term drug treatment, binge drinking, harm reduction variables

*SD* Standard deviation, *MDMA* Methylenedioxymethamphetamine, *LSD* Lysergic acid diethylamide, *NPSs* New psychoactive substances

^a^Occasional: up to 1 unit of alcohol per day, moderate: up to 2 units of alcohol per day for females and 3 units of alcohol per day for males, high: more than 2 units of alcohol per day for females and 3 units of alcohol per day for males

^b^6 or more drinks on one occasion

*Fisher’s exact test

Class 1 (53%, *N* = 163) was mainly represented by the use of ecstasy/MDMA or cocaine (probabilities of 42% and 22% in the previous year, respectively) and use of magic mushrooms (12%) and LSD (10%), albeit lower probabilities. Class 2 (47%, *N* = 151) was represented by higher probabilities of ecstasy/MDMA and cocaine use (79% and 83%, respectively) than Class 1 1 and LSD (35%) to a lesser extent. Other drugs were only reported in Class 2: ketamine (25%), speed (22%), and NPSs (13%).

The mean number of different drugs reported was more than twice as high in Class 2 than in Class 1 (3.9 versus 1.7). Compared to Class 1, participants in Class 2 more frequently attended dub and electronic music festivals (*p* = 0.034), and they were more likely to be unemployed (14.2%, *N* = 21 versus 5.7%, *N* = 9, *p* = 0.036). Tobacco and alcohol use was also more frequent in Class 2 than in Class 1 (*p* = 0.04 and *p* = 0.028, respectively), and binge drinking was more frequent (*p* < 0.001): twice as many participants in Class 2 reported binge drinking more than 4 times per month.

With regard to risk reduction, participants in Class 2 more frequently reported getting information about substances before using them (84.6%, *N* = 126 versus 75.2%, *N* = 118) (Table 3). The most frequently reported information topics spontaneously were: effects, risks, and the least frequently reported was: ways to limit risks. Most of the time, participants reported getting information from friends, peers, and less frequently from the Internet and associations. More than one in two persons reported that risk reduction materials and information were easily accessible. However, persons in Class 2 reported being more frequently aware of social actors and harm reduction and/or prevention structures (Table 3).

**Fig. 2:**
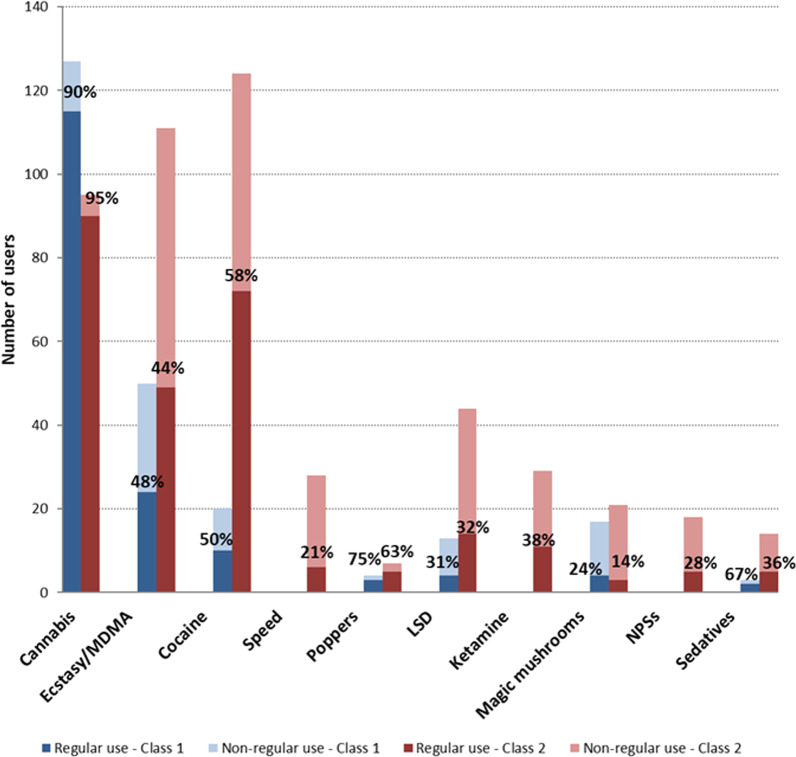
Distribution of regular use (at least monthly) of drugs according to the two latent classes. *MDMA* Methylenedioxymethamphetamine, *LSD* Lysergic acid diethylamide, *NPSs* New psychoactive substances. The height of the bars represents the number of persons who reported using the substance, and the proportion of persons with regular use is represented by the dark part of the bar. Missing values: 0–22% of missing values for each drug, 35% for poppers

The regularity of drug use (monthly use at least) was quite similar in the two classes: frequent use for the three most reported drugs (approximately 9/10 persons who use cannabis and approximately half of persons who use ecstasy/MDMA or cocaine); this was the same for poppers but with a far lower number of persons (Fig. 2).

Problematic use was assessed among persons with regular use for all substances and then by the most reported substances. The results showed a higher frequency of problematic use in Class 2 than in Class 1 for all substances (92%, *N* = 95/103 versus 65%, *N* = 74/114 Chi-square test *p* < 0.0001). The analysis for the most reported substances showed a higher frequency of problematic use in Class 2 than in Class 1 for cannabis (74%, *N* = 64/86 versus 55%, *N* = 59/108 Chi-square test *p* = 0.005), whereas it was not different for the most frequent stimulants ecstasy/MDMA and cocaine (combined frequency of problematic use among persons with regular use: 39% (12/31) versus 39% (42/107), respectively, Chi-square test *p* = 0.96).

## Discussion

In this large study on festival attendees, we found a high prevalence of drug use (more than 80%), and the most reported drugs were cannabis, ecstasy/MDMA and cocaine. Two profiles of illicit drug use were identified. Class 1 (half of persons who reported using drugs), which could be interpreted as “no/low polysubstance use”, was characterized mainly by the use of “classic” stimulants (ecstasy/MDMA and cocaine) and also hallucinogen use (LSD, magic mushrooms) in lower probabilities. Class 2 (half of persons who reported using drugs), which could be interpreted as “moderate/extensive polysubstance use”, was characterized by higher probabilities of “classic” drug use than in Class 1 and other substances reported only in Class 2: speed, ketamine, and NPSs. The number of drugs used was twice as high in Class 2 as in Class 1. The two classes differed mainly concerning nightlife habits (music type and tobacco and alcohol consumption), even though an association with employment status was also observed. Most participants who use substances reported getting information about substances before using them (even if more frequently in Class 2); however, ways to limit risks were the least common type of information mentioned. Social actors and harm reduction or prevention structures were frequently known by participants, although also more frequently in Class 2. However, it should be noted that associations and the Internet were cited much less than friends and peers as sources of information in both classes.

### A high prevalence of drug use

The high prevalence of drug use in our study population, with the most reported drugs being cannabis and stimulants such as ecstasy/MDMA and cocaine, is consistent with previous studies on the party scene, even if prevalence may vary slightly across countries and types of events [21, 22, 25, 26, 33]. Hallucinogens were relatively frequently reported in our study population (10–18% for LSD, ketamine and magic mushrooms). The importance of hallucinogens in the party scene is in the same range as other studies in the United States (15–17% in the electronic dance music scene) [11] and Australia (13–25% in mixed-genre live festivals) [22, 25], but an even higher prevalence is observed in the German party scene (33–35% for LSD and 20–50% for ketamine) [21, 28]. An increase in the availability and use of hallucinogens is observed in France and in all of Europe in recent years [5, 15]. In particular, ketamine is becoming “trendy” at parties, and it is mostly used for stimulating and inebriating effects with moderate use.

Other substances, such as inhaled substances (nitrous oxide, poppers) or GHB-GBL, were not frequently reported in this work, but their use is gradually increasing since 2017 in France [2]. The temporality of our study (2017–2018) and the various recreational settings may explain this finding. The prevalence of NPS use was not rare in our study, even if mentioned in the fewest substance categories (5% of persons who use drugs). The use of NPSs is highly variable between European countries [1], particularly in the UK [18], but it is also frequent in studies of electronic dance music scenes in Germany (20% of persons who use drugs) [28].

### Different profiles of drug use

We found two classes of drug use profiles, whereas 3–4 classes are found in previous studies using LCA of the party scene [13, 27–29]. This difference can be explained by (i) the methodology used (the population analysed: only persons who use drugs in our work versus the entire study population or the choice of the indicators) and (ii) the context: different countries, times or festive events. The class identified as polysubstance use in our work (Class 2, “moderate/extensive polysubstance use”) included more people than in the other studies (half of persons who use drugs versus 10–20%). Indeed, our Class 2 included persons with moderate and extensive polysubstance use (versus distinct profiles identified in previous studies) and showed less “severe” markers of polysubstance use (lower probabilities of drug use and number of drugs used). However, we found similar results according to the typology of drug use: moderate “classic drug” use (cannabis, ecstasy/MDMA, cocaine) in the “no or low polysubstance use” profile and higher probabilities of “classic substances” with other particular drugs (ketamine, NPSs, speed) in the “moderate/extensive polysubstance use” profile [13, 27–29]. These results highlight the use of ketamine, NPSs and speed by persons who consume “classic substances”. Indeed, persons who use NPS are found to have used a large variety of substances in their lifetime [6, 9, 10, 24, 34].

### Similarities between profiles

Despite the obvious differences in terms of the number and variety of drugs used in the two profiles, we have to emphasize that the regularity of use (at least monthly) was quite similar. This result could be surprising but we may raise the hypothesis of a profile of persons searching for new experiences using other substances in addition to the “classic” ones.

### Differences between profiles

Differences in characteristics between the two classes were mainly related to recreational nightlife habits: more frequently attending dub or electronic music events, higher frequency of tobacco and alcohol consumption and binge drinking in the “moderate/extensive polysubstance use” class compared to the “no/low polysubstance use” class. These findings are in accordance with those of Sanudo et al*.* and Fernandez-Calderon et al*.* regarding their “high level of polysubstance use” [13, 29], and we can hypothesize that it attests to a riskier type of substance use. The only associated sociodemographic characteristic was employment status (profile of “moderate/extensive polysubstance use” more likely to be unemployed); this association is already highlighted in extensive polysubstance use profiles [13]. However, we must note that our study population was mainly represented by persons with good social integration, a stable household and a high education level. We can hypothesize that the profile of “moderate/extensive polysubstance use” is in fact possibly composed of different populations. Indeed, the increase in the availability of substances (such as ketamine and LSD) in different social environments [2] and the normalization of drug use in the party scene [2, 6] may contribute in this way.

In addition, we must keep in mind that polysubstance use is associated with the risk of substance use disorders [35], and in fact, we observed a higher proportion of problematic use in the “moderate/extensive polysubstance use” profile than in the “no/low polysubstance use” profile. However, we have to emphasize that our study population was in its great majority without any reported medical history (low frequency of long-term diseases and long-term drug treatments).

### Challenges for risk reduction

Our results showed that most participants reported getting information about substances before using them in both classes (although more often in Class 2), but ways to limit risks were the least commonly reported type of information. Moreover, social actors and harm reduction and/or prevention structures were frequently known by participants, but the least cited sources of information. Regarding the high frequency of the polysubstance use profile, many participants may be concerned with limiting the risks associated with multiple substance use. Messages of risk reduction by social actors or structures should generally reinforce the risks of interactions between substances, which could lead to serious adverse events. According to our results, risk reduction messages can be targeted according to the profile of polysubstance use. For persons no or low polysubstance use, messages of prevention regarding “classic stimulants” (ecstasy/MDMA, cocaine and hallucinogens such as LSD and magic mushrooms) could be enhanced. For persons with higher polysubstance use, risk reduction messages could also be targeted regarding specific substances that were found only in this profile” (speed, ketamine, and NPSs). This focus is all the more important at certain types of music events, including electronic or dub music events, where we found that the profile of moderate/extensive polysubstance use was more frequent.

### Strengths and limitations

The main strength of this study is the study population of festival attendees included in a variety of music festivals, “hidden persons who use substances” who are difficult to reach in the research field. However, we should note that the size of the study population is quite moderate, impacting the generalizability of our results. The standardized data collection process by trained interviewers (volunteers trained in addictology or harm reduction strategies) ensures that the data are of good quality. Moreover, we analysed drug use in the previous year, allowing the study of all types of consumption, including nonparty consumption, and preventing seasonal variations. However, we have to emphasize some limitations. We chose a population of festival attendees because they are known to be particularly at risk for drug use. However, this type of study deserves to be replicated in other recreational settings, which can also be the place of choice for drug use [7]. We may have underestimated the use of psychoactive substances because of some well-known limitations related to self-reported data on substance use: social desirability bias (use of face-to-face interviews) or recall bias (analysis of use in the past year). However, we believe that the statuses of the interviewers (volunteers trained in addiction care or harm reduction strategies) may have limited the possibility of underreporting. On the other hand, we cannot exclude a selection bias, however participants were offered to participate at random and we did not have any refusals to participate. Furthermore, we cannot exclude that participants may have participated more than once, although the probability is very low. In addition, drugs were analysed as they were reported by the participants, but persons who use drugs cannot be certain of the exact content of the drugs used. For example, mephedrone was reported in our work but its sale is very restricted in France since 2010 [36]. These limitations are well known in drug surveys, which is why biological studies are complementary tools for monitoring drug use [7, 33]. To finish, it should be noted that our data pre-dates the COVID-19 pandemic, and we know that the health crisis has an impact on substance use [30].

## Conclusions

We found two profiles of drug use among festival attendees: a “no or low polysubstance use” profile with mainly “classic drug” use and a “moderate/extensive polysubstance use” profile with high probabilities of “classic substances” and also other particular drugs (ketamine, NPSs, speed). The “moderate/extensive polysubstance use” profile differed by a large variety of illicit drugs used and associated other types of consumption (tobacco, alcohol and especially binge drinking). Harm reduction strategies related to polysubstance use and the associated risk of toxicity should be strengthened for all persons who use drugs, and particularly for specific drugs (ketamine, NPSs, speed) for persons with heavy polysubstance use.

## Data Availability

The datasets used and/or analysed during the current study are available from the corresponding author on reasonable request.
